# Initiation, titration, and safety of vericiguat for treatment of heart failure in United States clinical practice

**DOI:** 10.1016/j.ahjo.2026.100721

**Published:** 2026-01-13

**Authors:** Stephen J. Greene, Alexander Michel, Coralie Lecomte, Paolo Manca, Katsiaryna Holl, Michele Senni

**Affiliations:** a Division of Cardiology, Duke University Medical Center/Duke Clinical Research Institute, Durham, USA; b Duke Clinical Research Institute, Durham, NC, USA; c Real World Evidence Centre of Excellence, Bayer Consumer Care AG, Basel, Switzerland; d Science, Aetion, a Datavant company, New York, USA; e Medical Department, Bayer S.p.A., Milano, Italy; f Global Medical & Evidence Cardiology, Bayer AG, Berlin, Germany; g Cardiovascular Department and Cardiology Unit, ASST Papa Giovanni XXIII, Bergamo, Italy

**Keywords:** Vericiguat, Titration patterns, Hypotension, Syncope, Real-world

## Abstract

**Study objective:**

To evaluate the following among new users of vericiguat: up-titration patterns, factors associated with up-titration, occurrence of hypotension/syncope, predictors of hypotension/syncope.

**Design:**

Retrospective cohort study (linked claims and electronic health record data).

**Setting:**

US clinical practice.

**Participants:**

1361 new users of vericiguat.

**Interventions:**

N/A.

**Main outcome measures:**

Vericiguat starting dose, up-titration patterns and predictors, occurrence and predictors of hypotension/syncope, over a 3-month follow-up period.

**Results:**

Among 1361 new users of vericiguat, 770 (57%) initiated a starting dose of 2.5 mg/day, 330 (24%) initiated a dose of 5 mg/day, and 261 (19%) initiated a dose of 10 mg/day. Over 3-month follow-up, the 10 mg target dose was reached by 349 (26%) patients. Among these patients, the median time to reach the 10 mg dose was 60 days among 2.5 mg/day starters, and 41 days among 5 mg/day starters. Among the 2.5 mg starters, 68% had no up-titration. Among patients initiating either the 2.5 mg/day or 5 mg/day dose, a starting dose of 5 mg (vs. 2.5 mg) was the only significant predictor for reaching the 10 mg dose; adjusted hazard ratio 2.89 (95% CI: 1.86, 4.49, *p* < 0.0001). Overall, 130 patients (9.6%) had a hypotension event and 67 patients (4.9%) had a syncope event. History of hypotension was the strongest independent predictor of hypotension/syncope events (adj. HR 2.85, 95% CI: 1.96, 4.13, *p* < 0.0001). A > 2.5 mg/day vericiguat starting dose was not associated with the occurrence of hypotension/syncope (vs. 2.5 mg/day); adj. HR 0.82, 95% C.I. (0.58, 1.16).

**Conclusion:**

Vericiguat users initiated on the 5 mg/day dose were considerably more likely to reach the target dose of 10 mg/day vs. those started on the recommended 2.5 mg/day dose, without excess risk of hypotension or syncope.

## Graphical abstract

**Figure:**
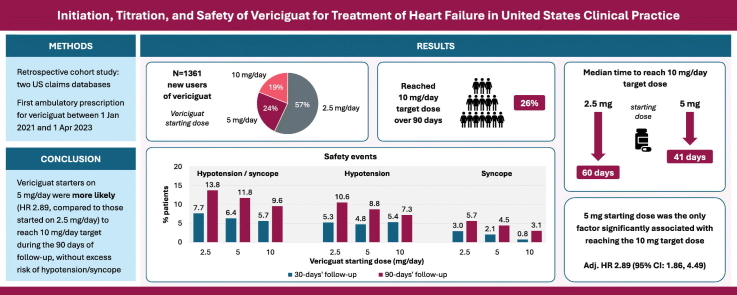

## 1 Introduction

Contemporary guidelines for heart failure with reduced ejection fraction (HFrEF) recommend the use of guideline directed medical therapy (GDMT) with titration to the maximally tolerated or target dose [1], [2]. However, data from real-world clinical practice consistently show suboptimal implementation of GDMT in terms of initiation and up-titration of therapy [3], [4]. Although medication intolerance, out-of-pocket costs, organizational aspects and other challenges can be barriers for some patients, the totality of evidence increasingly supports clinical inertia as the dominant reason for widespread systemic gaps in both older generic and newer branded GDMTs [5], [6]. Specifically, despite prognosis comparable to many forms of cancer, patients with heart failure (HF) might be deemed clinically stable on current therapy, with this stability misconstrued as low risk and a reason to defer escalation of life-prolonging therapy [5], [7], [8].

Vericiguat, a soluble guanylate cyclase stimulator available since 2021, is recommended in the European Society of Cardiology (ESC) guidelines for patients with chronic HFrEF and New York Heart Association class II–IV, who have had a worsening event despite GDMT to reduce the risk of cardiovascular mortality or heart failure hospitalization (HFH) [1]. The SOCRATES-REDUCED trial [9] suggested a dose-response relationship between increasing vericiguat dose and NT-proBNP reduction, and in the VICTORIA trial [10], 10 mg/day was the effective target dose tested. Current labeling recommends the initiation of vericiguat at 2.5 mg daily, increasing to 5 mg at 2 weeks and further increasing to 10 mg target dose at 4 weeks. In the VELOCITY study [7] initiation of vericiguat at a dose of 5 mg (rather than 2.5 mg) daily was safe and well-tolerated in >90% of 106 patients for whom this was undertaken, with minimal effects on systolic blood pressure and low rates of symptomatic hypotension. Such a one-step titration scheme could ultimately lead to more patients reaching the 10 mg target dose.

In a large cohort study from real-world practice, we evaluated whether a 5 mg starting dose could facilitate up-titration, and whether there could be a signal of increased hypotension risk associated with this higher dosage. The objectives were to evaluate up-titration patterns of vericiguat, factors associated with up-titration and the occurrence of, and predictors for hypotension or syncope.

## 2 Materials and methods

### 2.1 Data source and study population

This study utilized two large, closed claims data sources (PS17 and PS20 with over 120 million and 87 million patients, respectively) from HealthVerity™ Marketplace in the United States (US). The claims sources included medical and pharmacy claims with information on inpatient and outpatient diagnoses and procedures [11]. Claims were linked to electronic health records (EHRs) from Veradigm® consisting of de-identified real world data of patients drawn from physician practices using a variety of EHR products. These data provide clinical depth that is not available from claims data alone including vital statistics, patient history, and laboratory tests and results. For inclusion in the study cohort, patients were required to have ≥6 months continuous enrolment before the index date, a first vericiguat closed pharmacy claim during the patient identification period and aged ≥18 years or older at the index date. Because this study used prior collected and anonymized data, institutional review board approval was not required.

### 2.2 Vericiguat treatment

Exposure to vericiguat was identified using relevant treatment terminologies (i.e. generic name or World Health Organization Anatomical Therapeutic Chemical codes). All vericiguat prescriptions and dosages during the first 90 days following the index date were captured. The start of treatment was defined as the date of the first closed pharmacy claim for vericiguat during the identification period with no previous vericiguat exposure in the 180 days before the index date. The vericiguat treatment episode was defined as the total number of days supplied, encompassing the initial fill and any subsequent refills. A grace period of 30 days was permitted between fills; if no refill information was available, it was assumed that no further refills occurred. In case of overlapping fills on the same day (e.g. one fill for 2.5 mg and one fill for 5 mg on the same day) or overlapping treatment episodes, it was assumed that the episode durations were additive (i.e. the supply days of the second overlapping episode are shifted forward, as stockpiling was assumed).

### 2.3 Follow-up and safety outcomes

Cohort members were followed from the index date until the outcome of interest, vericiguat discontinuation, disenrollment, end of follow-up (1 July 2023), or death, whichever came first. With a last possible index date of 1 April 2023, this enabled for all patients a 90-day observation period after starting vericiguat. Safety outcomes of interest were hypotension and syncope. Hypotension events were identified by any medical claims or EHR record with a diagnosis code of hypotension (ICD-10: I95.0, I95.1, I95.2, I95.89, I95.9) or a documented systolic blood pressure < 90 mmHg. Syncope was identified by any medical claim or EMR with a diagnosis code of syncope (ICD-10: R55), excluding other syncope codes that specify a given etiology (e.g., heat syncope, carotid sinus syncope) (ICD-10: F48.8, G90.01, T67.1).

### 2.4 Covariates

Data were obtained on age at the index date, sex, and comorbidities, cardiac procedures, and GDMT during the baseline period (the 6 months before the index date). Comorbidities included hypertension, hyperlipidemia, ischemic heart disease (IHD), diabetes mellitus, chronic kidney disease, atrial fibrillation, anemia, and history of hypotension. We also identified patients with a previous HFH during the baseline period, which was defined as an inpatient claim with a heart failure diagnosis code and a duration of >1 day, as well as those with a previous worsening event was defined as a HFH during the baseline period or use of intravenous diuretics during the 90 days before the index date. Procedures included coronary artery bypass graft, percutaneous coronary intervention, biventricular pacemaker, and implantable cardioverter defibrillator. Guideline-directed medical therapies included beta-blockers, angiotensin-converting enzyme inhibitors (ACEis), angiotensin receptor blockers (ARBs), angiotensin receptor-neprilysin inhibitor (ARNi), mineralocorticoid receptor antagonists (MRAs), and sodium-glucose co-transporter 2 inhibitor (SGLT2is).

### 2.5 Statistical analysis

Baseline characteristics of the study cohort were summarized using counts and percentages for categorical variables and means with standard deviation (SD) or median with interquartile range (IQR) for continuous variables. Vericiguat treatment patterns were described including the starting dose, up-titration patterns, and time to reach daily dose. Multivariable Cox regression was used to identify factors associated with developing hypotension or syncope (individually and as a composite endpoint) as well as factors associated with reaching 10 mg/day at any time during the 3-month follow-up period among patients with a 2.5 mg or 5 mg first recorded dose. In the latter model, patients who had 10 mg as the first recorded dose were excluded. Candidate co-variates for the multivariable model were age, sex, comorbidities, cardiac procedures, and GDMT during the baseline period (the 6 months before the index date). Pearson correlation coefficient between the dependent variable and the pre-selected variables was estimated to assess collinearity. Collinear variables, defined as those with a correlation coefficient >0.8 or variance inflation factor >5 were dropped, and then the multivariable model refitted. Frequencies of hypotension and syncope (separately and as a composite endpoint) were assessed over the total 90-day follow-up period and separately for the first, second, and third month of follow-up. As treatment initiation during hospitalization could not be identified in the database, we repeated the analysis excluding patients with a HFH during the 4 weeks before the index date of the first ambulatory prescription. Furthermore, we stratified results according to presence/absence of a previous worsening event. All analyses were performed using Aetion® Substantiate and R version 4.2.3.

## 3 Results

### 3.1 Patient characteristics and starting dose of vericiguat

A total of 1361 patients initiated vericiguat and fulfilled the selection criteria for inclusion in this study. Of these, the dose of the index vericiguat prescription was 2.5 mg in 770 patients (57%), 5 mg in 330 patients (24%), and 10 mg in 261 patients (19%). Baseline demographics, clinical characteristics and medications are displayed in Table 1. Hypertension (90%), IHD (68%) and diabetes mellitus (61%) were among the most frequent comorbidities. Around 41% of patients had a history of a recent worsening HF event as defined previously. A history of hypotension was more frequent among patients starting on 2.5 mg vericiguat (18.3%) compared with those starting on 5 mg (13.6%) or 10 mg (14.6%). Regarding background therapy with GDMT, 60% of the cohort were prescribed ARNi and 45% were prescribed a SGLT2i. Roughly 20% were prescribed quadruple GDMT and 35% triple GDMT.

**Table 1:** Baseline characteristics (in the 6 months before the index date) by first recorded dose of vericiguat.

Vericiguat cohort	Total N = 1361	Starting dose 2.5 mg/day N = 770	Starting dose 5 mg/day N = 330	Starting dose 10 mg/day N = 261
**Age** at the index date, years	61.76 (14.27)	61.71 (14.34)	61.42 (14.06)	62.36 (14.37)
**Sex**				
Female	474 (34.8)	261 (33.9)	125 (37.9)	88 (33.7)
Male	887 (65.2)	509 (66.1)	205 (62.1)	173 (66.3)
**Comorbidities**^a^				
Anemia	533 (39.2)	294 (38.2)	131 (39.7)	108 (41.4)
Atrial fibrillation	437 (32.1)	245 (31.8)	116 (35.2)	76 (29.1)
CKD	478 (35.1)	270 (35.1)	114 (34.5)	94 (36.0)
Diabetes mellitus	833 (61.2)	491 (63.8)	192 (58.2)	150 (57.5)
Hyperlipidemia	1003 (73.7)	577 (74.9)	233 (70.6)	193 (73.9)
Ischemic heart disease	922 (67.7)	527 (68.4)	227 (68.8)	168 (64.4)
Hypertension	1230 (90.4)	705 (91.6)	293 (88.8)	232 (88.9)
History of hypotension	224 (16.5)	141 (18.3)	45 (13.6)	38 (14.6)
**Procedures**^a^				
CABG	78 (5.7)	42 (5.5)	21 (6.4)	15 (5.7)
PCI	60 (4.4)	34 (4.4)	12 (3.6)	14 (5.4)
Biventricular pacemaker	87 (6.4)	46 (6.0)	24 (7.3)	17 (6.5)
ICD	539 (39.6)	320 (41.6)	132 (40.0)	87 (33.3)
**Recent HF event**				
Recent HF hospitalization^a^	503 (37.0)	277 (36.0)	130 (39.4)	96 (36.8)
Recent worsening event^b^	554 (40.7)	307 (39.9)	144 (43.6)	103 (39.5)
**GDMT**^a^				
Beta blocker	1215 (89.3)	682 (88.6)	302 (91.5)	231 (88.5)
ACEi	312 (22.9)	176 (22.9)	76 (23.0)	60 (23.0)
ARB	307 (22.6)	175 (22.7)	75 (22.7)	57 (21.8)
ARNi	817 (60.0)	454 (59.0)	217 (65.8)	146 (55.9)
MRA	640 (47.0)	362 (47.0)	162 (49.1)	116 (44.4)
SGLT2i	605 (44.5)	342 (44.4)	141 (42.7)	122 (46.7)
**Number of GDMTs**^a^				
Monotherapy	169 (12.4)	94 (12.2)	41 (12.4)	34 (13.0)
Dual therapy	406 (29.8)	229 (29.7)	94 (28.5)	83 (31.8)
Triple therapy	475 (34.9)	267 (34.7)	121 (36.7)	87 (33.3)
Quadruple therapy	270 (19.8)	155 (20.1)	66 (20.0)	49 (18.8)

Data are n (%) or mean (±SD).

ACEi, angiotensin-converting enzyme inhibitor; ARB, angiotensin receptor blocker; ARNi, angiotensin receptor-neprilysin inhibitor; CABG, coronary artery bypass graft; CI, confidence interval; CKD, chronic kidney disease; GDMT, guideline-directed medical therapy; HFH, heart failure hospitalization; HR, hazard ratio; MRA, mineralocorticoid receptor antagonist; PCI, percutaneous coronary intervention; SD, standard deviation; SGLT2i, sodium-glucose co-transporter 2 inhibitor.

a During the baseline period (the 6 months before the index date).

b Defined as: HFH in the 180 days before the index date, or intravenous diuretics use in the 90 days before the index date.

### 3.2 Vericiguat titration patterns

**Fig. 1:**
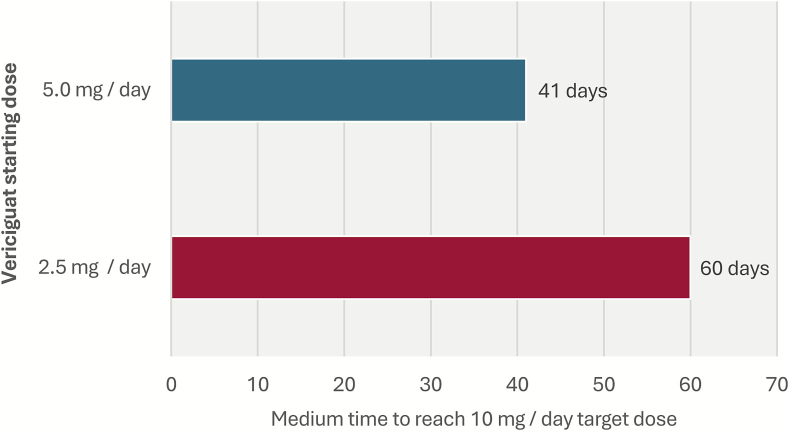
Time to reach 10 mg/day target dose among patients who reached the target dose and started with 2.5 or 5 mg/day.

Over half of the study cohort (56.6%) started on vericiguat at a daily dose of 2.5 mg/day, 24.2% started on 5 mg/day, and 19.2% on 10 mg/day. The observed vericiguat up-titration patterns are displayed in Table 2. Ninety-two percent of patients had the full 3 months of follow-up available. The 10 mg target dose was reached by 349 patients (26%) during the 3-month follow-up. Among those starting with 5 mg (*n* = 330), 15% reached the 10 mg dose, while among those starting with 2.5 mg, 5% reached the target dose. Among the 2.5 mg starters, 68% did not have any up-titration during the 3 months of follow-up period. The median time to reach the 10 mg dose was 60 days among those starting with 2.5 mg/day and 41 days for those starting with 5 mg/day (Fig. 1).

**Table 2:** Vericiguat up-titration patterns over 90-day follow-up.

Vericiguat daily dose at index date	Total 1361
2.5 mg/day	770 (56.6)
5 mg/day	330 (24.2)
10 mg/day	261 (19.2)
**Any up-titration after index date**	189 (13.9)
**Specific up-titration patterns**	
***Starting dose 2.5 mg***	
No further up-titration	523 (38.4)
Up-titration to 5 mg/day and no further up-titration	73 (5.4)
Up-titration to 5 mg/day, and then to 10 mg/day	22 (1.6)
Up-titration directly to 10 mg/day without 5 mg/day titration	13 (1.0)
***Starting dose 5 mg***	
No further up-titration	252 (18.5)
Up-titration to 10 mg/day	49 (3.6)
***Starting dose 10 mg***	
Remaining at 10 mg/day	250 (18.4)
**Vericiguat up-titration**	
**Patients reaching target dose across 90 days' follow-up**	349 (25.6)
**Time to reach first target dose across 90 days' follow-up**	
**2.5 mg starting dose**	
Mean (SD) days	56.85 (21.0)
Median (Q1, Q3) days	60 (42, 70)
**5 mg starting dose**	
Mean (±SD) days	46.98 (22.6)
Median (IQR) days	41 (29–62)

Data are n (%).

### 3.3 Factors associated with reaching the vericiguat 10 mg target dose

Among those starting with 2.5 mg or 5 mg, a starting dose of 5 mg (vs. 2.5 mg) was the only significant predictor for reaching the 10 mg dose; adj. HR 2.89 (95% CI: 1.86, 4.49, *p* < 0.0001; Table 3). This finding was consistent when stratifying the analysis according to presence/absence of a previous worsening event; adj. HR 2.27 (95% CI: 1.18, 4.39, *p* = 0.015) in patients with a previous worsening event, and adj. HR 3.25 (95% CI: 1.74, 6.06, *p* = 0.0002) in patients without a previous worsening event (Supplementary Tables 1 and 2).

**Table 3:** Predictors evaluated for reaching the target dose (10 mg) over 90 days among patients starting with 2.5 mg or 5 mg vericiguat (Cox regression model).

	Univariable HR (95% CI)	*p* value	Multivariable HR (95% CI)	*p* value
Age at the index date, years				
≥75	Ref		Ref	
65–74	0.74 (0.41, 1.34)	0.325	1.01 (0.45, 2.25)	0.982
50–64	1.34 (0.87, 2.06)	0.187	1.32 (0.66, 2.62)	0.433
<50	0.92 (0.52, 1.64)	0.777	1.13 (0.49, 2.62)	0.780
Sex				
Male	Ref		Ref	
Female	0.96 (0.60, 1.51)	0.850	1.02 (0.63, 1.65)	0.936
Vericiguat starting dose				
2.5 mg/day	Ref		Ref	
5 mg/day	3.13 (2.03, 4.82)	<0.0001	2.89 (1.86, 4.49)	<0.0001
Comorbidities^a^				
Anemia				
No	Ref		Ref	
Yes	1.18 (0.76, 1.83)	0.467	1.12 (0.68, 1.84)	0.654
CKD				
No	Ref		Ref	
Yes	1.52 (0.98, 2.34)	0.059	1.43 (0.86, 2.35)	0.166
Hypotension				
No	Ref		Ref	
Yes	1.03 (0.58, 1.82)	0.929	0.84 (0.45, 1.56)	0.589
Hypertension				
No	Ref		Ref	
Yes	0.91 (0.45, 1.81)	0.781	0.70 (0.32, 1.51)	0.362
Atrial fibrillation				
No	Ref		Ref	
Yes	1.18 (0.75, 1.85)	0.470	1.12 (0.68, 1.82)	0.657
Obesity				
No	Ref		Ref	
Yes	1.24 (0.80, 1.91)	0.336	1.05 (0.67, 1.67)	0.820
Devices and procedures^a^				
PCI				
No	Ref		Ref	
Yes	1.28 (0.47, 3.50)	0.629	1.41 (0.50, 3.98)	0.513
Biventricular pacemaker				
No	Ref		Ref	
Yes	0.79 (0.29, 2.16)	0.649	0.92 (0.31, 2.71)	0.875
ICD				
No	Ref		Ref	
Yes	0.63 (0.40, 1.01)	0.058	0.63 (0.37, 1.08)	0.093
GDMT^a^				
Number of GDMTs				
No/missing GDMT use	0.41 (0.06, 2.94)	0.375	0.69 (0.09, 5.55)	0.729
Monotherapy	Ref	Ref	Ref	Ref
Dual therapy	0.61 (0.36, 1.05)	0.073	0.80 (0.42, 1.53)	0.495
Triple therapy	1.38 (0.89, 2.13)	0.148	Not selected	NA
Quadruple therapy	1.40 (0.86, 2.28)	0.177	Not selected	NA
ARB/ACEi				
No	Ref	Ref	Ref	Ref
Yes	1.29 (0.84, 1.99)	0.239	1.31 (0.81, 2.10)	0.271
ARNi				
No	Ref	Ref	Ref	Ref
Yes	1.38 (0.87, 2.20)	0.173	1.33 (0.78, 2.24)	0.294
SGLT2i				
No	Ref	Ref	Ref	Ref
Yes	1.46 (0.95, 2.25)	0.084	1.18 (0.72, 1.94)	0.516
Beta blocker				
No	Ref		Ref	
Yes	2.27 (0.83, 6.19)	0.111	1.75 (0.61, 5.01)	0.298
MRA				
No	Ref		Ref	
Yes	1.34 (0.87, 2.07)	0.1849	1.21 (0.74, 2.00)	0.4469
Previous worsening event^b^				
No	Ref		Ref	
Yes	1.58 (1.03, 2.44)	0.036	1.23 (0.75, 2.04)	0.409

Patients were censored upon disenrollment, vericiguat discontinuation, or death.

ACEi, angiotensin-converting enzyme inhibitor; ARB, angiotensin receptor blocker; ARNi, angiotensin receptor-neprilysin inhibitor; CI, confidence interval; CKD, chronic kidney disease; GDMT, guideline-directed medical therapy; HR, hazard ratio; ICD, implantable cardioverter defibrillator; MRA, mineralocorticoid receptor antagonist; PCI, percutaneous coronary intervention; SGLT2i, sodium-glucose co-transporter 2 inhibitor.

a During the baseline period (the 6 months before the index date).

b Defined as: HFH in the 180 days before the index date, or intravenous diuretics use in the 90 days before the index date.

### 3.4 Safety events

**Fig. 2:**
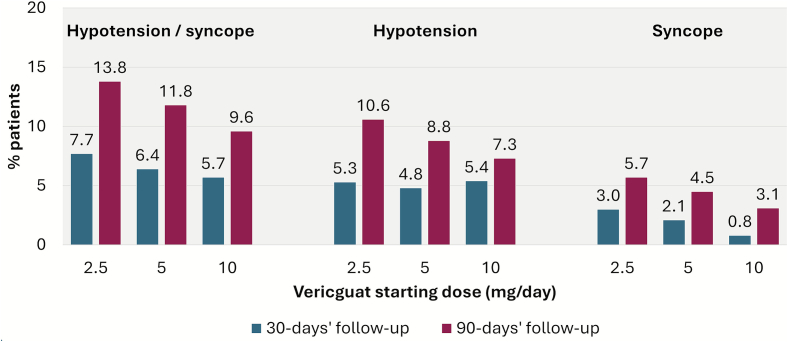
Frequency of hypotension and syncope during follow-up among patients who initiated vericiguat (*n* = 1361).

The frequency of hypotension and syncope events over the total 90-day follow-up period as well as during the first, second and third month of follow-up are displayed in Fig. 2. Overall, 130 patients (9.6%) had a hypotension event recorded and 67 patients (4.9%) had a syncope event during the 90-day follow-up. We observed no increase in frequency of hypotension and syncope events in patients starting on vericiguat dose of 5 or 10 mg/day relative to those starting on 2.5 mg/day. This pattern did not change when restricting to patients without a previous HFH the 4 weeks before the first vericiguat prescription (to avoid including patients who may have started during a hospital stay) (Supplementary Table 3). There was overall a higher frequency of hypotension/syncope in patients with a previous worsening event, compared to those without, but no positive association was seen between vericiguat dose and incidence of these events (Supplementary Table 4).

In the multivariable Cox model for hypotension/syncope (Table 4), a history of hypotension was the strongest predictor associated with hypotension/syncope events (adj. HR 2.85, 95% CI: 1.96, 4.13, *p* < 0.0001). Other predictors for hypotension/syncope were anemia (adj. HR 1.66, 95% CI: 1.16, 2.39, *p* = 0.006) and previous worsening HF event (adj. HR 1.67, 1.13, 2.48, *p* = 0.01). Baseline use of calcium channel blockers was independently associated with lower risk of hypotension/syncope (adj. HR 0.37, 95% CI: 0.19, 0.71, *p* = 0.003). There was no significant association between vericiguat starting dose of >2.5 mg (vs. 2.5 mg) and risk of hypotension/syncope (adj. HR 0.82, 95% CI: 0.58, 1.16, *p* = 0.26). These results were consistent among patients with a previous worsening event adj. HR (0.68, 95% CI: 0.42, 1.11, *p* = 0.12) and those without a previous worsening HF event (adj. HR 1.26, 95% CI: 0.74, 2.15, *p* = 0.4) (Supplementary Tables 5 and 6).

**Table 4:** Predictors examined for the development of hypotension/syncope over 90 days (Cox regression model) among all patients starting vericiguat.

	Univariable HR (95% CI)	*p* value	Multivariable HR (95% CI)	*p* value
Age at the index date, years				
≥75	Ref		Ref	
65–74	0.93 (0.61, 1.42)	0.731	0.85 (0.47, 1.53)	0.582
50–64	1.05 (0.76, 1.45)	0.782	0.90 (0.53, 1.51)	0.685
<50	1.11 (0.73, 1.69)	0.622	0.92 (0.48, 1.74)	0.792
Gender				
Male	Ref		Ref	
Female	1.16 (0.82, 1.62)	0.402	1.09 (0.76, 1.56)	0.639
Vericiguat starting dose				
2.5 mg/day	Ref		Ref	
>2.5 mg/day	0.75 (0.54, 1.06)	0.101	0.82 (0.58, 1.16)	0.259
Comorbidities^a^				
Atrial fibrillation				
No	Ref		Ref	
Yes	1.21 (0.86, 1.70)	0.265	0.98 (0.68, 1.41)	0.899
Anemia				
No	Ref		Ref	
Yes	1.75 (1.26, 2.43)	0.0008	1.66 (1.16, 2.39)	0.006
CKD stage 3 or higher				
No	Ref		Ref	
Yes	0.99 (0.62, 1.59)	0.971	0.76 (0.45, 1.28)	0.296
Myocardial infarction				
No	Ref		Ref	
Yes	1.48 (1.06, 2.06)	0.021	1.17 (0.81, 1.70)	0.406
Hypotension				
No	Ref		Ref	
Yes	3.61 (2.58, 5.05)	<0.0001	2.85 (1.96, 4.13)	<0.0001
Hypertension				
No	Ref		Ref	
Yes	0.86 (0.51, 1.44)	0.565	0.48 (0.26, 0.90)	0.021
Hyperlipidemia				
No	Ref		Ref	
Yes	1.14 (0.78, 1.67)	0.489	1.22 (0.77, 1.94)	0.391
Diabetes mellitus (type 1 and type 2)				
No	Ref		Ref	
Yes	1.04 (0.74, 1.45)	0.831	1.03 (0.70, 1.52)	0.867
Dementia				
No	Ref		Ref	
Yes	0.56 (0.18, 1.74)	0.314	0.35 (0.11, 1.18)	0.091
Obesity				
No	Ref		Ref	
Yes	0.96 (0.68, 1.34)	0.797	0.77 (0.53, 1.11)	0.156
Devices and procedures^a^				
PCI				
No	Ref		Ref	
Yes	0.81 (0.33, 1.99)	0.653	0.52 (0.20, 1.31)	0.165
Biventricular pacemaker				
No	Ref		Ref	
Yes	1.01 (0.51, 1.98)	0.986	0.82 (0.40, 1.68)	0.584
ICD				
No	Ref		Ref	
Yes	1.40 (1.01, 1.95)	0.042	1.13 (0.78, 1.64)	0.514
GDMT^a^				
Number of GDMTs for HF at baseline				
No/missing GDMT use	0.69 (0.22, 2.16)	0.521	0.61 (0.17, 2.16)	0.442
Monotherapy	Ref		Ref	
Dual therapy	0.78 (0.53, 1.14)	0.196	0.70 (0.42, 1.16)	0.161
Triple therapy	1.20 (0.86, 1.68)	0.284	0.91 (0.60, 1.37)	0.637
Quadruple therapy	0.99 (0.66, 1.49)	0.968	Not selected	n/a
Medication use^a^				
ACEi/ARB				
No	Ref		Ref	
Yes	1.13 (0.81, 1.57)	0.473	1.28 (0.89, 1.84)	0.188
ARNi				
No	Ref		Ref	
Yes	1.22 (0.87, 1.71)	0.258	1.48 (1.00, 2.19)	0.051
SGLT2i				
No	Ref		Ref	
Yes	0.84 (0.60, 1.17)	0.312	0.69 (0.47, 1.00)	0.053
Beta blocker				
No	Ref		Ref	
Yes	1.12 (0.64, 1.94)	0.699	0.90 (0.48, 1.69)	0.747
MRA				
No	Ref		Ref	
Yes	1.31 (0.94, 1.81)	0.109	0.92 (0.62, 1.35)	0.657
Comedications^a^				
Calcium channel blocker				
No	Ref		Ref	
Yes	0.33 (0.17, 0.63)	0.001	0.37 (0.19, 0.71)	0.003
Nitrate				
No	Ref		Ref	
Yes	1.42 (1.00, 2.03)	0.053	1.40 (0.96, 2.06)	0.082
Antidepressants				
No	Ref		Ref	
Yes	1.65 (1.18, 2.31)	0.003	1.36 (0.95, 1.95)	0.098
Previous worsening event^b^				
No	Ref		Ref	
Yes	2.21 (1.59, 3.08)	<0.0001	1.67 (1.13, 2.48)	0.010

Patients were censored upon disenrollment, vericiguat discontinuation, or death.

ACEi, angiotensin-converting enzyme inhibitor; ARB, angiotensin receptor blocker; ARNi, angiotensin receptor-neprilysin inhibitor; CI, confidence interval; CKD, chronic kidney disease; GDMT, guideline-directed medical therapy; HR, hazard ratio; ICD, implantable cardioverter defibrillator; MRA, mineralocorticoid receptor antagonist; PCI, percutaneous coronary intervention; SGLT2i, sodium-glucose co-transporter 2 inhibitor.

a During the baseline period (the 6 months before the index date).

b Defined as: HFH in the 180 days before the index date, or intravenous diuretics use in the 90 days before the index date.

## 4 Discussion

In this cohort of patients with HF initiated on vericiguat in US clinical practice, the large majority of patients are not up-titrated to the 10 mg target dose within 3 months of starting therapy.

Our findings are in line with other reports documenting minimal titration of GDMTs for HFrEF over longitudinal follow-up [3], [4] Likewise, in other recent real-world studies of vericiguat from Japan [12] and Germany [13], the percentage of patients reaching the target dose was 32% and 36%, respectively, slightly above the 26% observed in our study (median follow-up time was 150 days in the German study, while patients were followed for 90 days in the study from Japan, similar to our study). This is in contrast to the results from the VICTORIA and VICTOR clinical trials, where 89% and 85%, of patients, respectively, were up-titrated to the 10 mg target dose, as well as findings from two single-center, real-world observational studies from Spain, where a 70% rate for reaching the target dose was reported [14], [15], [16].

Reducing the number of medication titration steps could, at least partly, overcome clinical inertia and lead to more patients receiving the target dose. Results of our study support this concept, showing that patients starting with a 5 mg/day dosage, and thus omitting one titration step, were approximately three times more likely to reach the target dose of 10 mg vericiguat/day compared with those starting on 2.5 mg/day. Even for those reaching the target dose, there was a substantial delay, which could be improved by omitting one titration step. The recent VELOCITY trial [7] showed that >9 of 10 patients safely tolerated initiation of vericiguat at the 5 mg/day dose. This aligns with our finding of no safety signal for more hypotension events associated with the higher starting dose as well as with previous findings from the VICTORIA trial, showing overall only mild effects for vericiguat on blood pressure compared with placebo, even in vulnerable patient populations [17]. The strongest predictor for hypotension in our study was a previous history of hypotension; other predictors were anemia and having a recent HF worsening event. These general predictors were also identified in a large observational cohort study from the UK, examining risk factors for development of hypotension in patients with HF [18].

Relative to previous studies of patients with HF in US clinical practice, the current study population of patients initiating vericiguat was relatively well treated with high proportional use of ARNi, SGLT2is, triple or quadruple therapy. Forty three percent of patients had a first recorded vericiguat dose higher than 2.5 mg/day, and a similarly high percentage was recently reported from an analysis from another US claims database [19]. This high figure could partly be due to vericiguat initiation at 2.5 mg/day during a hospitalization, with the subsequent first outpatient prescription fill being 5 mg daily. To mitigate this possible bias in documenting the vericiguat starting dose, we undertook several sensitivity analyses, excluding all patients who had a HFH within the four weeks before the first ambulatory prescription, as well as excluding all patients with a HFH during the 6 months before (by stratifying according to previous worsening event). However, across these sensitivity analyses, we did not see any material change in the results. A further source of bias could have been that some patients might have received free samples of medication, which would not have been recorded in the database.

### 4.1 Limitations

Limitations of this study should be noted. With reliance on diagnosis codes, hypotension episodes were potentially under-recorded in this study and actual blood pressure data were not available for all patients (only 6–7% of hypotension events could be derived from actual blood pressure data). We adjusted the estimates for potential confounders at baseline; however, some clinically relevant information (e.g. on blood pressure, HF severity, left ventricular ejection fraction or NT-proBNP measurements) were not available for the majority of patients in the database and could have resulted in residual or unmeasured confounding.

## 5 Conclusions

In this nationwide analysis of vericiguat titration patterns in routine US clinical practice, initiation of vericiguat at a 5 mg starting dose (vs. a 2.5 mg starting dose) was associated with a greater likelihood (HR 2.89) of achieving target dosing without excess risk of hypotension or syncope. In conjunction with safety and tolerability data from vericiguat clinical trials, the current data could support a potential update in clinical guidance towards initiation of vericiguat with a 5 mg/day starting dose for select patients.

## Previous publication

While this work has not been published previously, some data from the study were presented at the ESC Heart Failure Congress 2025, 17–20 May, Belgrade, Serbia.

## CRediT authorship contribution statement

**Stephen J. Greene:** Writing – review & editing. **Alexander Michel:** Conceptualization, Methodology, Supervision, Writing – review & editing. **Coralie Lecomte:** Formal analysis, Investigation, Methodology, Project administration, Writing – review & editing. **Paolo Manca:** Conceptualization, Methodology, Writing – review & editing. **Katsiaryna Holl:** Conceptualization, Methodology, Writing – review & editing. **Michele Senni:** Writing – review & editing.

## Ethical considerations

This study used prior collected and anonymized data and institutional review board approval was not required.

## Funding

This work was supported by 10.13039/100004326Bayer AG. The sponsor (Bayer AG) was involved in the study design, the analysis and interpretation of data, reviewing manuscript drafts, and decision to submit the article for publication, as per the contributions of the Bayer Employee authors (Alexander Michel, Paolo Manca, and Katsiaryna Holl) as shown in the CRediT author statement at the end of the manuscript.

## Declaration of competing interest

Stephen J Greene has received research support from the American Heart Association, Amgen, AstraZeneca, Bayer, Boehringer Ingelheim, Bristol Myers Squibb, Cytokinetics, Merck, Novartis, Otsuka, Pfizer, and Sanofi; has served on advisory boards or as consultant for Amgen, AstraZeneca, Bayer, Boehringer Ingelheim, Bristol Myers Squibb, Chugai, Corcept Therapeutics, Corteria Pharmaceuticals, CSL Vifor, Cytokinetics, Idorsia, Lexicon, Lilly, Merck, Novo Nordisk, Otsuka, Roche Diagnostics, Sanofi, scPharmaceuticals, Sumitomo, Tricog Health, and Viatris; and has received speaker fees from AstraZeneca, Bayer, Boehringer Ingelheim, Cytokinetics, Lexicon, Novartis, Novo Nordisk, and Roche Diagnostics. All other authors report no conflicts. Alexander Michel, Paolo Manca, and Katsiaryna Holl are employees of Bayer. Coralie Lecomte is an employee of Aetion, Inc. and holds stock options of Aetion, Inc. Michele Senni has the following disclosures: Consultancy for Novonordisk, Bayer AG, Cardurion Pharmaceuticals, Boehinger Ingelheim, Amgen, Astrazeneca, Merck, MSD Italy, Reprieve Cardiovascular, CPC Clinical Research, Boot HC srl., IQVIA Ltd. UK. Advisory boards for Astrazeneca, Novonordisk, Bayer AG, Merck, Boehringer Ingelheim, Cardurion Pharmaceuticals, Amgen, Merck. Speaker Fees for Novonordisk, Merck, MSD Italy, Bayer AG, Astrazeneca, Cardurion Pharmaceuticals, Eli Lily Italia Spa, Il Pensiero Scientifico editore srl.

## Appendix A Supplementary data

Supplementary figures Image 1
