# Supplementary material for: Initiation, titration, and safety of vericiguat for treatment of heart failure in United States clinical practice
Source: Am Heart J Plus. 2026 Jan 13;62:100721. doi: 10.1016/j.ahjo.2026.100721 (PMC12830299; doi:10.1016/j.ahjo.2026.100721)
Supplement: Supplementary file 1 — Supplementary figures [file mmc1.pdf]

Supplementary Table 1. Time to target dose (Cox model) among patients starting with vericiguat 2.5 or 5 mg, **with a previous worsening event**[a].

	**Univariable HR (95% CI)**	***p*-value**	**Multivariable HR (95% CI)**	***p*-value**
**Age at the index date, years**				
≥75	ref	ref	ref	ref
65–74	0.70 (0.25, 1.97)	0.5035	0.81 (0.20, 3.25)	0.7651
50–64	0.97 (0.52, 1.81)	0.9243	1.18 (0.39, 3.51)	0.7725
<50	1.19 (0.58, 2.43)	0.6409	1.65 (0.48, 5.69)	0.4294
**Gender**				
Male	ref	ref	ref	ref
Female	0.89 (0.46, 1.70)	0.7178	1.02 (0.51, 2.06)	0.9488
**Vericiguat starting dose**				
2.5 mg/day	ref	ref	ref	ref
5 mg/day	2.70 (1.45, 5.02)	0.0017	2.27 (1.18, 4.39)	0.0145
**Comorbidities**[b]				
Anemia				
No	ref	ref	ref	ref
Yes	1.52 (0.81, 2.86)	0.1874	1.99 (0.99, 4.00)	0.0517
Chronic kidney disease				
No	ref	ref	ref	ref
Yes	1.14 (0.60, 2.14)	0.6902	1.32 (0.64, 2.69)	0.4532
Hypotension				
No	ref	ref	ref	ref
Yes	0.78 (0.38, 1.59)	0.4873	0.79 (0.37, 1.72)	0.5561
Hypertension				
No	ref	ref	ref	ref
Yes	1.36 (0.19, 9.91)	0.7606	1.08 (0.14, 8.58)	0.9420
Atrial fibrillation				
No	ref	ref	ref	ref
Yes	1.13 (0.61, 2.12)	0.6948	1.36 (0.64, 2.89)	0.4187
Obesity				
No	ref	ref	ref	ref
Yes	0.79 (0.42, 1.47)	0.4506	0.64 (0.33, 1.24)	0.1834
**Devices and procedures**[b]				
Percutaneous coronary intervention				

	Univariable HR (95% CI)	*p*-value	Multivariable HR (95% CI)	*p*-value
No	ref	ref	ref	ref
Yes	0.85 (0.21, 3.54)	0.8276	0.92 (0.19, 4.37)	0.9178
Biventricular pacemaker				
No	ref	ref	ref	ref
Yes	0.31 (0.04, 2.25)	0.2460	0.35 (0.04, 2.89)	0.3313
ICD				
No	ref	ref	ref	ref
Yes	0.55 (0.28, 1.10)	0.0926	0.56 (0.25, 1.23)	0.1463
**GDMT[b]**				
**Number of GDMT (90 days prior CED incl.)**				
No/missing GDMT use	0.00000011 (0.00, Inf)	0.9967	0.00000016 (0.00, Inf)	0.9966
Monotherapy	ref	ref	ref	ref
Dual therapy	0.80 (0.38, 1.68)	0.5565	Not selected	n/a
Triple therapy	1.07 (0.57, 2.00)	0.8334	Not selected	n/a
Quadruple therapy	1.69 (0.86, 3.33)	0.1271	Not selected	n/a
**ARB/ACEi**				
No	ref	ref	ref	ref
Yes	1.34 (0.72, 2.50)	0.3539	1.44 (0.72, 2.86)	0.3031
**Use of ARNi**				
No	ref	ref	ref	ref
Yes	2.13 (1.04, 4.37)	0.0381	2.25 (1.01, 5.01)	0.0469
**SGLT2i**				
No	ref	ref	ref	ref
Yes	1.83 (0.97, 3.44)	0.0614	1.55 (0.79, 3.06)	0.2040
**Beta blcoker**				
No	ref	ref	ref	ref
Yes	3.46 (0.48, 25.20)	0.2202	2.33 (0.30, 18.42)	0.4218
**Use of MRA**				
No	ref	ref	ref	ref
Yes	1.35 (0.71, 2.55)	0.3641	1.33 (0.67, 2.64)	0.4152

[a] Defined as a HFH during the baseline period or use of intravenous diuretics during the 90 days before the index date.
[b] During the baseline period (the 6 months before the index date).
ACEi, angiotensin-converting enzyme inhibitor; ARB, angiotensin receptor blocker; ARNi, angiotensin receptor-neprilysin inhibitor; CABG, coronary artery bypass graft; CI, confidence interval; CKD, chronic kidney disease; GDMT, guideline-directed medical therapy; HFH, heart failure hospitalization; HR, hazard ratio; MRA, mineralocorticoid receptor antagonist;

PCI, percutaneous coronary intervention; SD, standard deviation; SGLT2i, sodium-glucose co-transporter 2 inhibitor

Supplementary Table 2. Time to target dose (Cox model) among patients starting with vericiguat 2.5 or 5 mg, **without a previous worsening event[a]**.

	Univariable HR (95 CI)	*p*-value	Multivariable HR (95 CI)	*p*-value
**Age at the index date**				
≥75	ref	ref	ref	ref
65–74	0.85 (0.41, 1.78)	0.6684	1.07 (0.39, 2.98)	0.8948
50–64	1.63 (0.89, 2.96)	0.1119	1.42 (0.55, 3.63)	0.4663
<50	0.55 (0.20, 1.54)	0.2528	0.61 (0.16, 2.31)	0.4683
**Sex**				
Male	ref	ref	ref	ref
Female	0.96 (0.50, 1.84)	0.8964	1.04 (0.52, 2.07)	0.9133
**Vericiguat starting dose**				
2.5 mg/day	ref	ref	ref	ref
5 mg/day	3.48 (1.90, 6.35)	0.0001	3.25 (1.74, 6.06)	0.0002
**Comorbidities[b]**				
Anemia				
No	ref	ref	ref	ref
Yes	0.73 (0.36, 1.48)	0.3861	0.58 (0.26, 1.27)	0.1710
Chronic kidney disease				
No	ref	ref	ref	ref
Yes	1.55 (0.80, 3.02)	0.1967	1.73 (0.82, 3.63)	0.1489
Hypotension				
No	ref	ref	ref	ref
Yes	1.10 (0.39, 3.08)	0.8574	1.12 (0.37, 3.38)	0.8424
Hypertension				
No	ref	ref	ref	ref
Yes	0.69 (0.32, 1.50)	0.3511	0.70 (0.28, 1.72)	0.4350
Atrial fibrillation				
No	ref	ref	ref	ref
Yes	1.09 (0.57, 2.10)	0.7861	1.04 (0.52, 2.11)	0.9074
Obesity				
No	ref	ref	ref	ref
Yes	1.67 (0.91, 3.04)	0.0964	1.74 (0.90, 3.35)	0.0978
**Device and procedures[b]**				
Percutaneous coronary intervention				
No	ref	ref	ref	ref

	Univariable HR (95 CI)	*p*-value	Multivariable HR (95 CI)	*p*-value
Yes	1.80 (0.44, 7.46)	0.4152	1.97 (0.42, 9.16)	0.3896
Biventricular pacemaker				
No	ref	ref	ref	ref
Yes	1.41 (0.44, 4.56)	0.5661	1.54 (0.42, 5.68)	0.5155
ICD				
No	ref	ref	ref	ref
Yes	0.72 (0.38, 1.36)	0.3109	0.68 (0.32, 1.42)	0.3018
**GDMT[b]**				
**Number of GDMT (90 days prior CED incl.)**				
No/missing GDMT use	0.62 (0.09, 4.53)	0.6400	0.64 (0.07, 5.92)	0.6921
Monotherapy	ref	ref	ref	ref
Dual therapy	0.50 (0.23, 1.09)	0.0807	0.45 (0.18, 1.13)	0.0877
Triple therapy	1.62 (0.89, 2.96)	0.1156	Not selected	n/a
Quadruple therapy	1.17 (0.58, 2.37)	0.6656	Not selected	n/a
**Use of ARB/ACEi**				
No	ref	ref	ref	ref
Yes	1.15 (0.63, 2.11)	0.6502	1.08 (0.53, 2.19)	0.8273
**ARNi**				
No	ref	ref	ref	ref
Yes	1.00 (0.54, 1.85)	0.9972	0.88 (0.42, 1.85)	0.7354
**SGLT2i**				
No	ref	ref	ref	ref
Yes	1.20 (0.66, 2.18)	0.5501	0.88 (0.44, 1.76)	0.7154
**Beta blocker**				
No	ref	ref	ref	ref
Yes	1.74 (0.54, 5.63)	0.3544	1.73 (0.47, 6.33)	0.4083
**MRA**				
No	ref	ref	ref	ref
Yes	1.22 (0.67, 2.22)	0.5123	0.97 (0.48, 1.94)	0.9244

[a] Defined as a HFH during the baseline period or use of intravenous diuretics during the 90 days before the index date.

[b] During the baseline period (the 6 months before the index date).

ACEi, angiotensin-converting enzyme inhibitor; ARB, angiotensin receptor blocker; ARNi, angiotensin receptor-neprilysin inhibitor; CABG, coronary artery bypass graft; CI, confidence interval; CKD, chronic kidney disease; GDMT, guideline-directed medical therapy; HFH, heart failure hospitalization; HR, hazard ratio; MRA, mineralocorticoid receptor antagonist;

PCI, percutaneous coronary intervention; SD, standard deviation; SGLT2i, sodium-glucose co-transporter 2 inhibitor

Supplementary Table 3. Frequency of hypotension and syncope during follow-up (patients with a HFH within 4 weeks before the index date were excluded).

Safety event	Starting dose 2.5 mg (N=653)	Starting dose 5 mg (N=282)	Starting dose 10 mg (N=234)
**Hypotension**			
Within 90 days' follow-up (first event)	52 (8.0)	19 (6.7)	16 (6.8)
Within 0–30 days of follow-up	20 (3.1)	8 (2.8)	12 (5.1)
Within 31–60 days' follow-up	15 (2.3)	7 (2.5)	8 (3.4)
Within 61–90 days' follow-up	23 (3.5)	9 (3.2)	4 (1.7)
**Syncope**			
Within 90 days' follow-up (first event)	31 (4.7)	12 (4.3)	8 (3.4)
Within 0–30 days' follow-up	16 (2.5)	4 (1.4)	2 (0.9)
Within 31–60 days' follow-up	15 (2.3)	3 (1.1)	3 (1.3)
Within 61–90 days' follow-up	8 (1.2)	7 (2.5)	3 (1.3)
**Hypotension/syncope**			
Within across 90 days' follow-up	71 (10.9)	28 (9.9)	22 (9.4)
Within 0–30 days' follow-up	33 (5.1)	12 (4.3)	13 (5.6)
Within 31–60 days' follow-up	28 (4.3)	8 (2.8)	10 (4.3)
Within 61–90 days' follow-up	27 (4.1)	16 (5.7)	7 (3.0)

Data are n (%)

Supplementary Table 4. Incidence of hypotension and syncope, stratified by presence/absence of a previous worsening event and starting dose.

	**Total**		**Starting dose 2.5 mg**		**Starting dose 5 mg**		**Starting dose 10 mg**	
	**Previous worsening event[a] (=554)**	**No previous worsening event [a] (N=807)**	**2 - Previous worsening event [a] (N=307)**	**No previous worsening event [a] (N=463)**	**Previous worsening event [a] (N=144)**	**- No previous worsening event [a] (N=186)**	**Previous worsening event [a] (N=103)**	**- No previous worsening event [a] (N=158)**
**Hypotension**	81 (14.6)	49 (6.1)	52 (16.9)	30 (6.5)	18 (12.5)	11 (5.9)	11 (10.7)	8 (5.1)
**Syncope**	35 (6.3)	32 (4.0)	27 (8.8)	17 (3.7)	6 (4.2)	9 (4.8)	2 (1.9)	6 (3.8)
**Hypotension or syncope**	98 (17.7)	72 (8.9)	65 (21.2)	41 (8.9)	20 (13.9)	19 (10.2)	13 (12.6)	12 (7.6)

Data are n (%)

[a] Defined as a HFH during the baseline period or use of intravenous diuretics during the 90 days before the index date.

Supplementary Table 5. Time to hypotension/syncope (Cox model), in patients **with a previous worsening event**[a].

	**Univariable HR (95% CI)**	***P* value**	**Multivariable HR (95% CI)**	***P* value**
**Age at the index date, years**				
≥75	ref	ref	ref	ref
65–74	0.87 (0.45, 1.68)	0.6715	0.50 (0.19, 1.30)	0.1573
50-64	1.05 (0.68, 1.62)	0.8283	0.67 (0.32, 1.42)	0.2983
<50	1.10 (0.66, 1.83)	0.7176	0.53 (0.22, 1.28)	0.1603
**Sex**				
Male	ref	ref	ref	ref
Female	1.18 (0.76, 1.82)	0.4638	0.93 (0.57, 1.51)	0.7668
**Vericiguat starting dose**				
2.5 mg/day	ref	ref	ref	ref
>2.5 mg/day	0.58 (0.37, 0.92)	0.0198	0.68 (0.42, 1.11)	0.1211
**Comorbidities**[b]				
Atrial Fibrillation				
No	ref	ref	ref	ref
Yes	0.88 (0.57, 1.38)	0.5869	0.80 (0.49, 1.29)	0.3527
Anemia				
No	ref	ref	ref	ref
Yes	1.31 (0.85, 2.02)	0.2266	1.62 (1.01, 2.60)	0.0456
CKD stage 3 or higher				
No	ref	ref	ref	ref
Yes	0.57 (0.29, 1.11)	0.0968	0.52 (0.25, 1.07)	0.0755
Myocardial infarction				
No	ref	ref	ref	ref
Yes	1.02 (0.67, 1.57)	0.9170	1.22 (0.75, 1.98)	0.4219
Hypotension				
No	ref	ref	ref	ref
Yes	2.53 (1.64, 3.89)	<0.0001	2.53 (1.60, 4.01)	<0.0001
Hypertension				
No	ref	ref	ref	ref
Yes	0.26 (0.12, 0.54)	0.0003	0.28 (0.12, 0.65)	0.0034
Hyperlipidemia				
No	ref	ref	ref	ref
Yes	0.63 (0.39, 1.02)	0.0595	0.85 (0.48, 1.50)	0.5669

	Univariable HR (95% CI)	*P* value	Multivariable HR (95% CI)	*P* value
Diabetes mellitus (type 1 and type 2)				
No	ref	ref	ref	ref
Yes	0.91 (0.58, 1.44)	0.6921	1.24 (0.71, 2.14)	0.4479
Dementia				
No	ref	ref	ref	ref
Yes	0.000000039 (0.00, Inf)	0.9950	0.000000014 (0.00, Inf)	0.9954
Obesity				
No	ref	ref	ref	ref
Yes	0.93 (0.61, 1.44)	0.7580	0.86 (0.53, 1.38)	0.5266
**Devices and procedures[b]**				
**PCI**				
No	ref	ref	ref	ref
Yes	0.52 (0.16, 1.65)	0.2667	0.48 (0.15, 1.57)	0.2244
**Biventricular pacemaker**				
No	ref	ref	ref	ref
Yes	1.07 (0.49, 2.32)	0.8607	0.82 (0.35, 1.91)	0.643
**ICD**				
No	ref	ref	ref	ref
Yes	1.59 (1.04, 2.45)	0.0335	1.32 (0.79, 2.20)	0.2938
**GDMTs**				
**Number of GDMT (90 days prior CED incl.)**				
No/missing GDMT use	1.74 (0.55, 5.52)	0.3460	0.98 (0.24, 3.96)	0.9738
Monotherapy	ref	ref	ref	ref
Dual therapy	0.71 (0.42, 1.19)	0.1949	0.79 (0.43, 1.43)	0.4279
Triple therapy	1.29 (0.83, 1.98)	0.2534	Not selected	n/a
Quadruple therapy	0.86 (0.49, 1.50)	0.5943	Not selected	n/a
**ACEi/ARB**				
No	ref	ref	ref	ref
Yes	1.05 (0.68, 1.61)	0.8340	1.34 (0.83, 2.17)	0.2279
**ARNi**				
No	ref	ref	ref	ref
Yes	1.10 (0.71, 1.72)	0.6632	1.29 (0.76, 2.19)	0.337
**SGLT2i**				
No	ref	ref	ref	ref

	**Univariable HR (95% CI)**	***P* value**	**Multivariable HR (95% CI)**	***P* value**
Yes	0.76 (0.49, 1.18)	0.2212	0.68 (0.41, 1.12)	0.1281
**Beta blocker**				
No	ref	ref	ref	ref
Yes	0.54 (0.29, 1.02)	0.0566	0.50 (0.23, 1.09)	0.0801
**MRA**				
No	ref	ref	ref	ref
Yes	1.31 (0.85, 2.03)	0.2252	1.04 (0.60, 1.78)	0.9006
**Comedications**				
**Calcium channel blocker**				
No	ref	ref	ref	ref
Yes	0.16 (0.05, 0.52)	0.0020	0.21 (0.06, 0.69)	0.0099
**Nitrate**				
No	ref	ref	ref	ref
Yes	1.24 (0.78, 1.96)	0.3715	1.44 (0.85, 2.43)	0.1720
**Antidepressant**				
No	ref	ref	ref	ref
Yes	1.75 (1.14, 2.70)	0.0107	1.63 (1.03, 2.59)	0.0370

[a] Defined as a HFH during the baseline period or use of intravenous diuretics during the 90 days before the index date

[b] During the baseline period (the 6 months before the index date)

ACEi, angiotensin-converting enzyme inhibitor; ARB, angiotensin receptor blocker; ARNi, angiotensin receptor-neprilysin inhibitor; CI, confidence interval; CKD, chronic kidney disease; GDMT, guideline-directed medical therapy; HR, hazard ratio; ICD, implantable cardioverter defibrillator; MRA, mineralocorticoid receptor antagonist; PCI, percutaneous coronary intervention; SGLT2i, sodium-glucose co-transporter 2 inhibitor

Supplementary Table 6. Time to hypotension/syncope (Cox model,) patients **without a previous worsening event**[a].

	**Univariable HR (95 CI)**	***p*value**	**Multivariable HR (95 CI)**	***p*value**
**Age at the index date, years**				
≥75	ref	ref	ref	ref
65–74	1.21 (0.68, 2.13)	0.5190	1.20 (0.56, 2.57)	0.6452
50–64	0.87 (0.52, 1.46)	0.6004	1.18 (0.56, 2.51)	0.6659
<50	0.87 (0.41, 1.83)	0.7128	1.34 (0.50, 3.61)	0.5662
**Sex**				
Male	ref	ref	ref	ref
Female	1.02 (0.60, 1.75)	0.9285	1.28 (0.73, 2.25)	0.3887
**Vericiguat starting dose**				
2.5 mg/day	ref	ref	ref	ref
>2.5 mg/day	1.01 (0.61, 1.68)	0.9602	1.26 (0.74, 2.15)	0.3998
**Comorbidities**[b]				
Atrial fibrillation				
No	ref	ref	ref	ref
Yes	1.45 (0.86, 2.46)	0.1663	1.15 (0.65, 2.04)	0.6373
Anemia				
No	ref	ref	ref	ref
Yes	1.85 (1.12, 3.07)	0.0171	1.48 (0.84, 2.63)	0.178
CKD stage 3 or higher				
No	ref	ref	ref	ref
Yes	1.66 (0.84, 3.27)	0.1431	1.68 (0.75, 3.75)	0.2087
Myocardial infarction				
No	ref	ref	ref	ref
Yes	1.47 (0.84, 2.57)	0.1774	1.09 (0.57, 2.06)	0.7958
Hypotension				
No	ref	ref	ref	ref
Yes	4.37 (2.44, 7.83)	<0.0001	3.75 (1.98, 7.10)	<0.0001
Hypertension				
No	ref	ref	ref	ref
Yes	1.08 (0.52, 2.28)	0.8304	0.50 (0.20, 1.23)	0.1307
Hyperlipidemia				
No	ref	ref	ref	ref
Yes	2.01 (1.05, 3.87)	0.0356	2.95 (1.27, 6.86)	0.0122
Diabetes mellitus (type 1 and type 2)				
No	ref	ref	ref	ref

	**Univariable HR (95 CI)**	***p*value**	**Multivariable HR (95 CI)**	***p*value**
Yes	0.98 (0.59, 1.62)	0.9263	0.72 (0.40, 1.30)	0.2767
Dementia				
No	ref	ref	ref	ref
Yes	1.43 (0.45, 4.58)	0.5425	1.12 (0.31, 4.05)	0.867
Obesity				
No	ref	ref	ref	ref
Yes	0.74 (0.42, 1.29)	0.2907	0.56 (0.30, 1.03)	0.0631
**Devices and procedures**[b]				
PCI				
No	ref	ref	ref	ref
Yes	1.22 (0.30, 4.99)	0.7829	0.85 (0.18, 4.02)	0.8369
Biventricular pacemaker				
No	ref	ref	ref	ref
Yes	0.61 (0.15, 2.51)	0.4954	0.82 (0.19, 3.58)	0.7863
ICD				
No	ref	ref	ref	ref
Yes	1.17 (0.71, 1.95)	0.5367	0.95 (0.54, 1.67)	0.8673
**GDMTs**				
**Number of GDMT (90 days prior CED incl.)**				
No/missing GDMT use	0.000000039 (0.00, Inf)	0.9959	0.000000065 (0.00, Inf)	0.9944
Monotherapy	ref	ref	ref	ref
Dual therapy	0.92 (0.53, 1.60)	0.7791	0.44 (0.20, 0.96)	0.0380
Triple therapy	0.95 (0.55, 1.64)	0.8579	0.59 (0.31, 1.14)	0.1162
Quadruple therapy	1.17 (0.65, 2.13)	0.5991	Not selected	n/a
**ACEi/ARB**				
No	ref	ref	ref	ref
Yes	1.09 (0.65, 1.81)	0.7498	1.21 (0.67, 2.16)	0.5335
**ARNi**				
No	ref	ref	ref	ref
Yes	1.43 (0.83, 2.47)	0.1926	1.45 (0.78, 2.72)	0.2401
**SGLT2i**				
No	ref	ref	ref	ref
Yes	0.89 (0.54, 1.49)	0.6629	0.70 (0.38, 1.28)	0.2447
**Beta blocker**				
No	ref	ref	ref	ref
Yes	2.80 (0.88, 8.95)	0.0816	2.90 (0.82, 10.19)	0.0974

	**Univariable HR (95 CI)**	***p*value**	**Multivariable HR (95 CI)**	***p*value**
**MRA**				
No	ref	ref	ref	ref
Yes	1.11 (0.67, 1.84)	0.6812	0.80 (0.43, 1.46)	0.4582
**Comedications[b]**				
**Calcium channel blocker**				
No	ref	ref	ref	ref
Yes	0.57 (0.26, 1.26)	0.1671	0.52 (0.23, 1.18)	0.1168
**Nitrates**				
No	ref	ref	ref	ref
Yes	1.49 (0.85, 2.61)	0.1630	1.43 (0.78, 2.62)	0.2457
**Antidepressants**				
No	ref	ref	ref	ref
Yes	1.27 (0.73, 2.20)	0.4005	1.13 (0.61, 2.07)	0.6997

[a] Defined as a HFH during the baseline period or use of intravenous diuretics during the 90 days before the index date

[b] During the baseline period (the 6 months before the index date)

ACEi, angiotensin-converting enzyme inhibitor; ARB, angiotensin receptor blocker; ARNi, angiotensin receptor-neprilysin inhibitor; CI, confidence interval; CKD, chronic kidney disease; GDMT, guideline-directed medical therapy; HR, hazard ratio; ICD, implantable cardioverter defibrillator; MRA, mineralocorticoid receptor antagonist; PCI, percutaneous coronary intervention; SGLT2i, sodium-glucose co-transporter 2 inhibitor
